# Identification of a Novel HIF-1α-α_M_β_2_ Integrin-NET Axis in Fibrotic Interstitial Lung Disease

**DOI:** 10.3389/fimmu.2020.02190

**Published:** 2020-10-15

**Authors:** Akif A. Khawaja, Deborah L. W. Chong, Jagdeep Sahota, Theresia A. Mikolasch, Charis Pericleous, Vera M. Ripoll, Helen L. Booth, Saif Khan, Manuel Rodriguez-Justo, Ian P. Giles, Joanna C. Porter

**Affiliations:** ^1^Centre for Inflammation and Tissue Repair, University College London, London, United Kingdom; ^2^Centre for Rheumatology, University College London, London, United Kingdom; ^3^Interstitial Lung Disease Service, University College London Hospital NHS Foundation Trust, London, United Kingdom; ^4^National Heart and Lung Institute, Imperial College London, London, United Kingdom; ^5^Institute of Nuclear Medicine, University College London, London, United Kingdom; ^6^Department of Histopathology, University College London Hospital NHS Foundation Trust, London, United Kingdom

**Keywords:** neutrophil, NET, hypoxia, HIF, endothelium, integrin, interstitial lung disease

## Abstract

Neutrophilic inflammation correlates with mortality in fibrotic interstitial lung disease (ILD) particularly in the most severe form, idiopathic pulmonary fibrosis (IPF), although the underlying mechanisms remain unclear. Neutrophil function is modulated by numerous factors, including integrin activation, inflammatory cytokines and hypoxia. Hypoxia has an important role in inflammation and may also contribute to pulmonary disease. We aimed to determine how neutrophil activation occurs in ILD and the relative importance of hypoxia. Using lung biopsies and bronchoalveolar lavage (BAL) fluid from ILD patients we investigated the extent of hypoxia and neutrophil activation in ILD lungs. Then we used *ex vivo* neutrophils isolated from healthy volunteers and BAL from patients with ILD and non-ILD controls to further investigate aberrant neutrophil activation in hypoxia and ILD. We demonstrate for the first time using intracellular staining, HIF-1α stabilization in neutrophils and endothelial cells in ILD lung biopsies. Hypoxia enhanced both spontaneous (+1.31-fold, *p* < 0.05) and phorbol 12-myristate 13-acetate (PMA)-induced (+1.65-fold, *p* < 0.001) neutrophil extracellular trap (NET) release, neutrophil adhesion (+8.8-fold, <0.05), and trans-endothelial migration (+1.9-fold, *p* < 0.05). Hypoxia also increased neutrophil expression of the α_M_ (+3.1-fold, *p* < 0.001) and α_X_ (+1.6-fold, *p* < 0.01) integrin subunits. Interestingly, NET formation was induced by α_M_β_2_ integrin activation and prevented by cation chelation. Finally, we observed NET-like structures in IPF lung sections and in the BAL from ILD patients, and quantification showed increased cell-free DNA content (+5.5-fold, *p* < 0.01) and MPO-citrullinated histone H3 complexes (+21.9-fold, *p* < 0.01) in BAL from ILD patients compared to non-ILD controls. In conclusion, HIF-1α upregulation may augment neutrophil recruitment and activation within the lung interstitium through activation of β_2_ integrins. Our results identify a novel HIF-1α- α_M_β_2_ integrin axis in NET formation for future exploration in therapeutic approaches to fibrotic ILD.

## Introduction

The interstitial lung diseases (ILD) are a group of diffuse parenchymal lung disorders that can result in pulmonary fibrosis (PF) (1). Despite recent advances in diagnostics and therapeutics, ILD is still associated with substantial morbidity and mortality (2). Neutrophil activation may be important in ILD, particularly the most severe fibrotic form, idiopathic PF (IPF). The pathogenesis of IPF is unknown but is thought to involve a “frustrated repair” response to repetitive epithelial injury, with associations to genes and proteins linked to epithelial function, integrity and repair. Progressive epithelial damage, and abnormal wound repair leads to extensive scar formation and correlates, clinically, with worsening hypoxia. Increasing desaturation during exercise (3) or sleep (4) is a significant predictor of mortality. Further evidence from animal models suggests that hypoxia may actually contribute to a vicious cycle of disease progression (5). This evidence has led to the view that hypoxia itself may contribute to worsening of PF but the mechanistic pathway is unknown.

Hypoxia, a state in which oxygen supply is inadequate for tissue demands, modulates gene expression via transcriptions factors called hypoxia inducible factors (HIF). There are 3 members of the HIF family, HIF-1α, HIF-2α, and HIF-3α, which bind conserved DNA sequences or Hypoxia Response Elements (HRE). Although it seems plausible that the IPF lung is hypoxic much of the evidence is indirect. Levels of lactic acid, a metabolite generated in response to hypoxia, are high in IPF lung tissue supporting the concept of a hypoxic microenvironment (6) and HIF-1α and -2α have been shown, *ex vivo*, to be expressed in lung biopsies from patients with IPF, in some but not all reports (7, 8). Additional genomic studies in IPF patients show up-regulation of hypoxia-related gene signatures, including TGF-β (9), the key fibrotic cytokine in PF, and of the HIF-1α pathways (8, 10).

The contribution of neutrophils to ILD has also been relatively less studied compared to other inflammatory and fibrotic diseases. Early studies began to explore the potential role of neutrophils in IPF (11–14), however research focus has since shifted to other cell types. The number of neutrophils in the bronchoalveolar lavage (BAL) fluid has been shown to predict both disease severity in IPF (15) and the development of PF in patients with hypersensitivity pneumonitis (16). In addition, neutrophil extracellular traps (NETs) have been shown to indirectly drive PF by stimulation of collagen production from fibroblasts *in vitro* (17), and NET release has been associated with PF in older mice *in vivo* (18) with loss of peptidyl arginine deiminase (PAD)-4, a key neutrophil enzyme for NET formation, being protective (18). Neutrophils are also associated with disease severity in acute lung injury and acute respiratory distress syndrome (ARDS) (19, 20) however, their precise contribution remains uncertain (21). Neutrophil depletion can ameliorate disease features in mouse models of ARDS (22) and a reduction in neutrophil infiltration (23), or knock-down of neutrophil elastase (NE) attenuates fibrosis in bleomycin-induced mouse models of PF (24). Taken together, these studies implicate a contributory role of neutrophils to fibrotic ILD.

Neutrophil survival is a tightly regulated process. Prolonged survival can delay resolution of inflammation and can cause damage to surrounding cells and tissues; however, if apoptosis is premature, antimicrobial function can be compromised (25). Hypoxia drives neutrophil survival via HIF-1α-dependent NF-κB activation (26). In addition, HIF-2α has also been shown to be important in regulating neutrophil function (27). Few reports address the effects of hypoxia upon NET formation. Inhibition of HIF-1α can reduce NET release (28), whilst pharmacological stabilization of HIF-1α increases phagocyte bactericidal activity (29) and NET release (30), implicating a role for down-stream targets of HIF-1α in leukocyte function.

Given the importance of hypoxia and HIF signaling in neutrophil function and the emerging role of neutrophils as key drivers of ILD, we sought evidence for hypoxia and NETs in the lungs of patients with ILD and the functional effects of low oxygen levels upon *ex vivo* neutrophil function and activation.

## Materials and Methods

### Bronchoalveolar Lavage

Fiber-optic bronchoscopy with BAL was performed in line with the American Thoracic Society guidelines (31). BAL was frozen for later analysis. None of the patients undergoing bronchoscopy had any infections at the time of procedure.

### Patient Demographics

BAL was obtained from 11 patients with fibrotic ILD and seven non-ILD controls undergoing diagnostic bronchoscopies. Demographics, clinical history and treatments at the time of sample collection are listed in Table 1. Within the ILD cohort: 4 (36%) had IPF, 3 (27%) had nonspecific interstitial pneumonia, 3 (27%) had chronic hypersensitivity pneumonitis (HP) and 1 (10%) had unclassifiable ILD. Our non-ILD control group underwent diagnostic bronchoscopy due to: 5 (71%) investigation of haemoptysis, 1 (14.5%) right middle lobe collapse and 1 (14.5%) previous tracheal schwannoma patients undergoing yearly bronchial surveillance. Only the ILD group had lung function tests, as part of standard patient care. None of the patients recruited were taking anti-fibrotic drugs at the time of bronchoscopy. Differential cell counts obtained from BAL from patients are listed in Supplementary Table 1.

**Table 1:** Patient clinical and demographic data.

		**ILD**	**Non-ILD Control**
Cohort size (*n*)		11	7
Age (years ±SD)		69 ± 5.9	51 ± 10.1
Sex (M:F)		8:3	4:3
Smoking Status (current:ever:never)		2:6:3	1:2:4
**Clinical Features**			
Diagnosis		IPF (4), fibrotic NSIP (3), chronic HP (3), unclassifiable ILD (1)	Haemoptysis (5), RML collapse (1), tracheal schwannoma (1)
Forced Vital Capacity (FVC) (liters) (mean ± SD) [% predicted]		2.7 ± 0.9 [80.4 ± 17.6%]	–
O_2_ saturation (mean ± SD)		96.2 ± 1.7%	–
TLCO (mmol/min/kPa) (mean ± SD)		50.8 ± 12.3	–
**Current Medications**			
Corticosteroids	Inhaled	Budesonide/formoterol fumarate dihydrate (1), fluticasone propionate/salmeterol xinafoate (1)	Budesonide/formoterol fumarate dihydrate (1)
	Oral	Methyl-prednisolone (1), prednisolone (1)	Prednisolone (1)
Nonsteroidal anti-inflammatory drugs		Aspirin (4)	Naproxen (1)

*Clinical details were recorded for all subjects at the time of bronchoalveolar lavage. Our ILD cohort had a mean age of 69 ± 5.9 years and consisted of four patients with IPF, three patients with fibrotic NSIP, three patients with chronic HP and one patient with unclassifiable ILD. Our non-ILD control group had a mean age of 51 ± 10.1 years and consisted of five patients with haemoptysis, one patient with RML collapse and one patient with tracheal schwannoma. Lung function was only obtained for the ILD cohort as part of standard care. FVC, forced vital capacity; HP, hypersensitivity pneumonitis; ILD, interstitial lung disease; IPF, idiopathic pulmonary fibrosis; NSIP, nonspecific interstitial pneumonia; RML, right middle lobe; TLCO, transfer factor for carbon monoxide*.

### Immunohistochemistry (IHC)

Lung biopsies were collected as part of routine clinical care. Ethical approval was given by the UK National Research Ethics Committee (13/LO/0900). IHC was performed using the automated Bond-Max system (Leica Biosystems Ltd., Newcastle) with 4 μm FFPE sections. HIF-1α (clone EP1215Y, Abcam, 1:600 dilution), myeloperoxidase (MPO) (polyclonal, Dako, 1:300 dilution) or NE (clone NP57, Dako, 1:100 dilution) was incubated in Epitope Retrieval Solution 2 for 20 min and stained using the 30, 20, 20 protocol. Test antibodies were controlled for using species- and isotype-matched control antibodies. Slides were scanned on a Nanozoomer Digital Slide Scanner and images analyzed using NDP viewer software (Hamamatsu Corportation). A “blinded” reviewer analyzed five randomly selected areas from each subject. Representative images were chosen from those selected.

### Neutrophil Isolation

Neutrophils were isolated as previously described (32). In brief, neutrophils were isolated by Percoll PLUS density centrifugation from sodium citrate anticoagulated blood obtained by informed consent from healthy volunteers. Neutrophils were diluted to 2 ×10^6^ neutrophils/ml in phenol-free RPMI (Thermo Scientific, UK) supplemented with 10% heat-inactivated FBS (Thermo Scientific, UK) and 2 mM L-gluatamine (Lonza, UK). To induce hypoxia, neutrophils were cultured under 1% oxygen in a Coy oxygen control glove box (Coy Laboratory Products Inc., USA) in a temperature controlled and humidified incubator.

### Endothelial Cell Culture

Human umbilical cord vein endothelial cells (HUVEC) (Lonza, Switzerland) were cultured in endothelial growth media 2 (Lonza, Switzerland) supplemented with 10% FBS (Thermo Scientific, UK) and 2 mM L-glutamine (Lonza, Switzerland) and used at passage 5. For endothelial activation, HUVEC were treated with 10 ng/ml TNF-α (R&D Systems, UK) for 24 h prior to experimentation. To induce hypoxia, HUVEC were cultured under 1% oxygen in a Coy oxygen control glove box (Coy Laboratory Products Inc., USA) in a temperature controlled and humidified incubator.

### Hydrogen Peroxide Generation

H_2_O_2_ generation was measured as previously described (32). In brief, neutrophils were cultured under normoxia or hypoxia for 1 h before addition of HRP (Sigma, UK) and Amplex^®^ UltraRed (Invitrogen, UK). H_2_O_2_ generation in response to phorbol 12-myristate 13-acetate (PMA) (Sigma, UK) was recorded using a FLUOstar Omega microplate reader (BMG Labetech, Germany) and rates (expressed in nM/sec) determined using Omega Mars Analysis software (BMG Labtech, Germany).

### NET Quantification

NETs were quantified using the Quanti-iT™ PicoGreen^®^ dsDNA kit (Invitrogen, UK) and using a capture ELISA. Streptavidin-coated plates (Fisher Scientific, UK) were coated with an anti-MPO capture antibody (Abcam, UK) overnight at 4° C and blocked with 0.5% bovine serum albumin for 1 h at 37° C. Neutrophil supernatants were incubated for 2 h at 37° C. Further 1 h incubations were performed with an anti-citrullinated histone H3 detection antibody (Abcam, UK) and HRP-conjugated secondary antibody (Dako, UK). SureBlue TMB Microwell Peroxidase Substrate (KPL, UK) was then added and incubated in the dark at 37° C for 20 min and then stopped by the addition of TMB stop solution (KPL, UK). Absorbance was read at 450 nm using a Tecan GENios Spectra FLUOR plate reader (Tecan UK Ltd., UK).

### NET Immunofluorescence

NETs were stained for immunofluorescence microscopy as described (32) using methodology modified from (33). In brief, 5 ×10^5^ neutrophils were added to fibrinogen-coated coverslips, stimulated for 4 h with 40 nM PMA, 0.5 mM MnCl_2_ or varying concentrations of leukadherin-1 (LA-1; Sigma, UK), and fixed with 4% PFA. Coverslips were blocked and sequentially incubated with an anti-histone H3 antibody (Abcam, UK) and Alexa Fluor^®^ 488-conjugated goat anti-rabbit IgG secondary antibody (Life Technologies, UK). Coverslips were washed, mounted, and sealed using with ProLong™ Gold antifade mountant with DAPI (Invitrogen, UK). Slides were visualized using a Zeiss Axio Imager.A1 inverted fluorescence microscope (Zeiss, Germany) and images analyzed using Image J.

### Lung Tissue Confocal Immunofluorescence

Lung sections were stained using a modified protocol based on published reports (34, 35). Five micrometer sections from paraffin-embedded lung biopsies from control or IPF patients were dewaxed prior to heat-induced epitope retrieval with Tris-EDTA buffer, pH 9.0. Sections were blocked with Fc block (BD biosciences, UK) before incubation with a blocking buffer (5% goat serum/2.5% BSA/PBS/0.1% Tween-20) for 1 h. Slides were then washed and incubated with anti-citrullinated histone H3 (Abcam, UK) and anti-MPO (R&D systems, UK) antibodies diluted in 0.5x blocking buffer overnight at 4° C. Anti-rabbit Alexa Fluor^®^ 647-conjugated and anti-mouse Alexa Fluor^®^ 555-conjugated secondary antibodies (Invitrogen, UK) and DAPI (Sigma, UK) diluted in 0.5x blocking buffer were then added for 30 min. Stained sections were washed, mounted, sealed and visualized using an Olympus inverted fluorescence confocal microscope and analyzed using Fluoviewer software (Olympus).

### BAL Confocal Immunofluorescence

BAL fluid was filtered using a 40 μm cell sieve. BAL cells were pelleted, counted and 1 ×10^5^ viable cells were used to produce cytospin slides (Thermo Shandon Cytospin 3, Thermo Scientific). Cytospin slides were fixed in 4% PFA, washed, and blocked overnight in blocking solution (10% goat serum/1% BSA/2 mM EDTA/HBSS/0.1% Tween-20). Slides were then washed and incubated with anti-histone H2A.X antibody (Abcam, UK) for 1 h before washing. Anti-rabbit Alexa Fluor^®^ 488-conjugated secondary antibody (Invitrogen, UK) and DAPI (Sigma, UK) were then diluted in blocking buffer for 1 h. Stained slides were then washed, mounted, sealed and visualized using an Olympus inverted fluorescence confocal microscope and analyzed using Fluoviewer software (Olympus).

### Neutrophil Integrin Expression

Cell surface expression of neutrophil integrins was evaluated by flow cytometry. Following isolation and culture under either normoxia or hypoxia, neutrophils were washed and resuspended in a sodium HEPES buffer (20 mM HEPES, 140 mM NaCl, 2 mg/ml glucose, 0.3% BSA). Cells were then stained using integrin subunit specific antibodies or appropriate isotype control for 30 min at room temperature. Stained cells were then washed twice, fixed in 2% PFA and assessed using a FACS Verse (BD Biosciences, UK). Data was analyzed using FlowJo (TreeStar Inc., UK).

### Neutrophil Adhesion

HUVEC were cultured in 96-well black tissue culture plates (Thermo Scientific, UK). Twenty-four hours prior to experimentation, HUVEC were subjected to normoxia or hypoxia in the absence or presence of 10 ng/ml TNF-α. Neutrophil adhesion in response to 20 nM PMA or 100 ng/ml lipopolysaccharide (LPS) were measured as previously described (32). Briefly, neutrophils were cultured under normoxia or hypoxia for 1 h, labeled with 2′,7′-bis-(2-carboxyethyl)-5-(and-6)-carboxyfluoresceinacetoxymethyl ester (Life Technologies, UK) and then added to wells under normoxia or hypoxia. Fluorescence was measured using a Tecan GENios Spectra FLUOR plate reader (Tecan UK Ltd., UK). Adhesion was calculated by comparing the fluorescence of washed wells to initial fluorescence.

### Neutrophil *trans*-Endothelial Migration

Trans-endothelial migration assays were performed as previously described (32). In brief, HUVEC were grown on transwell inserts (Millipore, UK). Twenty-four hours prior to experimentation, HUVEC were cultured under normoxia or hypoxia in the absence or presence of 10 ng/ml TNF-α. Neutrophils were cultured under normoxia or hypoxia for 1 h and then labeled with CellTracker (Invitrogen, UK). 1 × 10^6^ neutrophils were added to the upper chamber of transwells and allowed to migrate in the absence or presence of 150 ng/ml IL-8 in the lower chamber for 90 min. Percent transmigration was calculated by comparing the number of cells in the lower chamber and the number of neutrophils added to the upper chamber.

### Western Blotting

Cell lysates (10 μg protein) were resolved by electrophoresis and transferred to a polyvinylidene fluoride membrane (GE Healthcare, UK). Membranes were blocked for 1 h in 5% skimmed milk/TBS/0.1% Tween-20 and incubated with primary antibodies (1:1,000 dilution) overnight at 4°C. Membranes were then washed, incubated with HRP-conjugated secondary antibodies, and visualized using the Luminata Western HRP substrate system (Millipore, Ireland).

### Statistical Analysis

Data were evaluated using GraphPad Prism. Data were tested for normality using a Kolmogorov-Smirnov test. In experimental data sets only comparing two groups, a Mann-Whitney test was performed or a Wilcoxon matched pairs test. In data sets with two variables, data were assessed by two-way ANOVA with a Dunnet's or Sidak's multiple comparison test. Correlations were determined by two-tailed Pearson correlation coefficients. A *p* value below 0.05 was considered significant.

## Results

### Neutrophils and Endothelial Cells Stain Positive for HIF-1α in the ILD Lung

**Figure 1:**
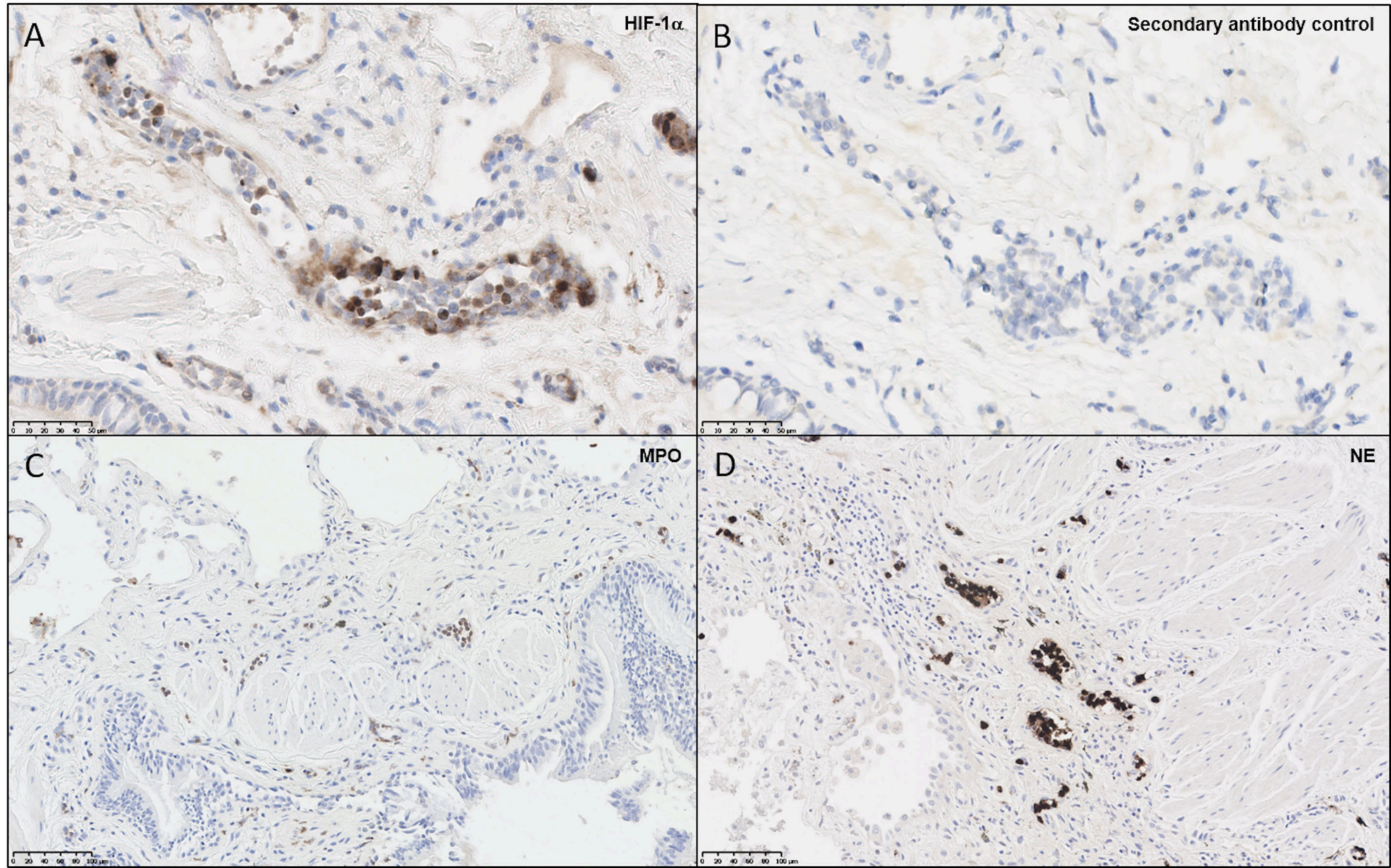
Neutrophils and endothelial cells express HIF-1α within the ILD lung. Paraffin-embedded from four lung biopsies from patients with fibrotic ILD, taken to determine clinical diagnosis of etiology, were cut and stained for immunohistochemical evidence of hypoxia and neutrophil infiltration. Images shown are representative of the four patients, from a patient diagnosed with IPF. **(A)** Slides stained for HIF-1α displayed positive brown staining within microvascular endothelial cells and polymorphonuclear cells, whilst **(B)** secondary antibody controls did not display positive staining. To verify whether neutrophils were present in the ILD lung, additional stains were performed for **(C)** MPO and **(D)** NE, both of which displayed positive brown stains within blood vessels. HIF-1α, hypoxia-inducible factor 1α; MPO, myeloperoxidase; NE, neutrophil elastase.

Given reports of localized hypoxia in pulmonary disease (36), biopsies from four patients with fibrotic ILD, performed to determine a clinical diagnosis of etiology, were examined for evidence of hypoxia. In this representative patient, diagnosed with IPF, HIF-1α staining demonstrated positive staining in the endothelium and polymorphonuclear cells, with very little staining in the fibrotic interstitium and overlying epithelium and no staining in control sections (Figures 1A,B). As aberrant NET formation has been implicated in several immunopathologies, we also stained lung sections for MPO and NE (Figures 1C,D), highlighting the presence of neutrophils within the pulmonary vasculature. Taken together, this staining pattern suggests that tissue-specific hypoxia and neutrophil recruitment may be a feature of the ILD lung. These findings led us to examine the effects of hypoxia upon neutrophil function.

### Hypoxic Exposure Does Not Affect Hydrogen Peroxide Generation but Promotes NET Release

**Figure 2:**
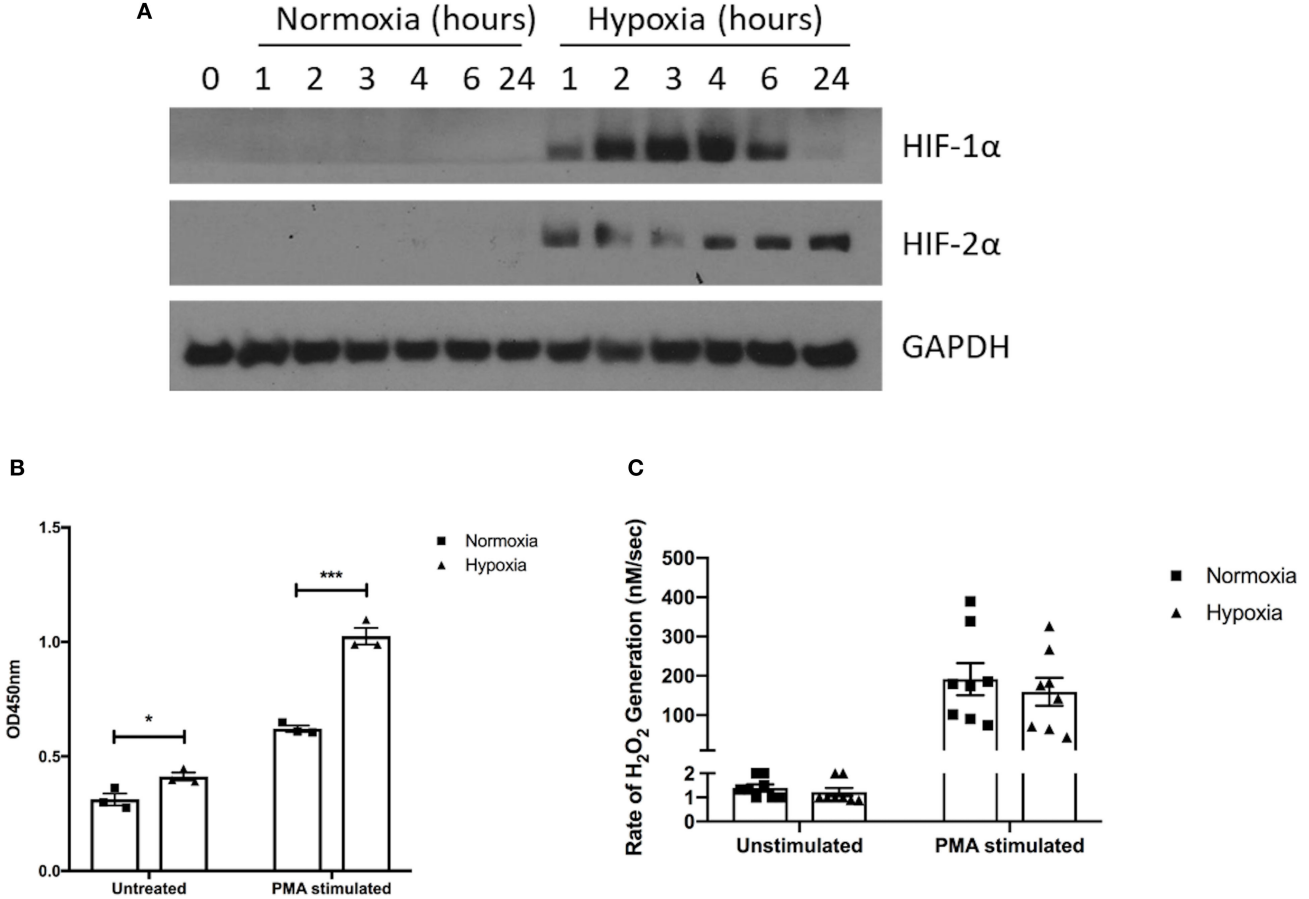
Hypoxia enhances NET release but not hydrogen peroxide production. The effects of hypoxia upon neutrophil activation was first assessed. **(A)** The induction of hypoxia was verified by Western blot, probing for HIF-1α and HIF-2α. **(B)** NET formation was then evaluated by capture ELISA, which detects MPO-citrullinated histone H3 complexes. Data are presented as the mean ±SEM from three different donors and analyzed by two-way ANOVA with a Dunnett's multiple comparison test. **(C)** Hydrogen peroxide generation was examined using Amplex^®^ UltraRed in absence and presence of 50 nM PMA. Data are presented as the mean ± SEM from neutrophils isolated from seven different donors and analyzed by two-way ANOVA with a Dunnett's multiple comparison test. ^*^ = *p* < 0.05, ^***^ = *p* < 0.001. HIF, hypoxia-inducible factor; PMA, phorbol 12-myristate 13-acetate.

Pharmacological HIF-1α stabilization has been reported to enhance bacterial killing and NET release (28–30), however, these studies were performed using atmospheric oxygen levels. We therefore assessed for any alteration in function, described below, of healthy neutrophils under normoxia (21% oxygen) and hypoxia (1% oxygen). First, we verified hypoxia by examining neutrophil cell lysates for the presence of HIF-1α and HIF-2α. We observed rapid stabilization of HIF-1α under hypoxia, with delayed HIF-2α stabilization (Figure 2A).

Having demonstrated induction of hypoxia, we assessed neutrophil supernatants for MPO-citrullinated histone H3 complexes, which are specific for NETs. Hypoxic neutrophils displayed greater levels of both spontaneous (+1.31-fold, *p* < 0.05) and PMA-induced (+1.65-fold, *p* < 0.001) NET release (Figure 2B). As reactive oxygen species generation is thought to drive NET formation (37, 38), we also examined hydrogen peroxidase (H_2_O_2_) production. Rates of H_2_O_2_ generation however, were comparable between oxygen states for both unstimulated (1.4 ± 0.1 nM/s vs. 1.2 ± 0.2 nM/s) and PMA-stimulated (191.7 ± 40.83 nM/s vs. 159.3 ± 35.51 nM/s) neutrophils (Figure 2C).

### Neutrophil Adhesion and *trans*-Endothelial Migration Are Enhanced Under Hypoxia

**Figure 3:**
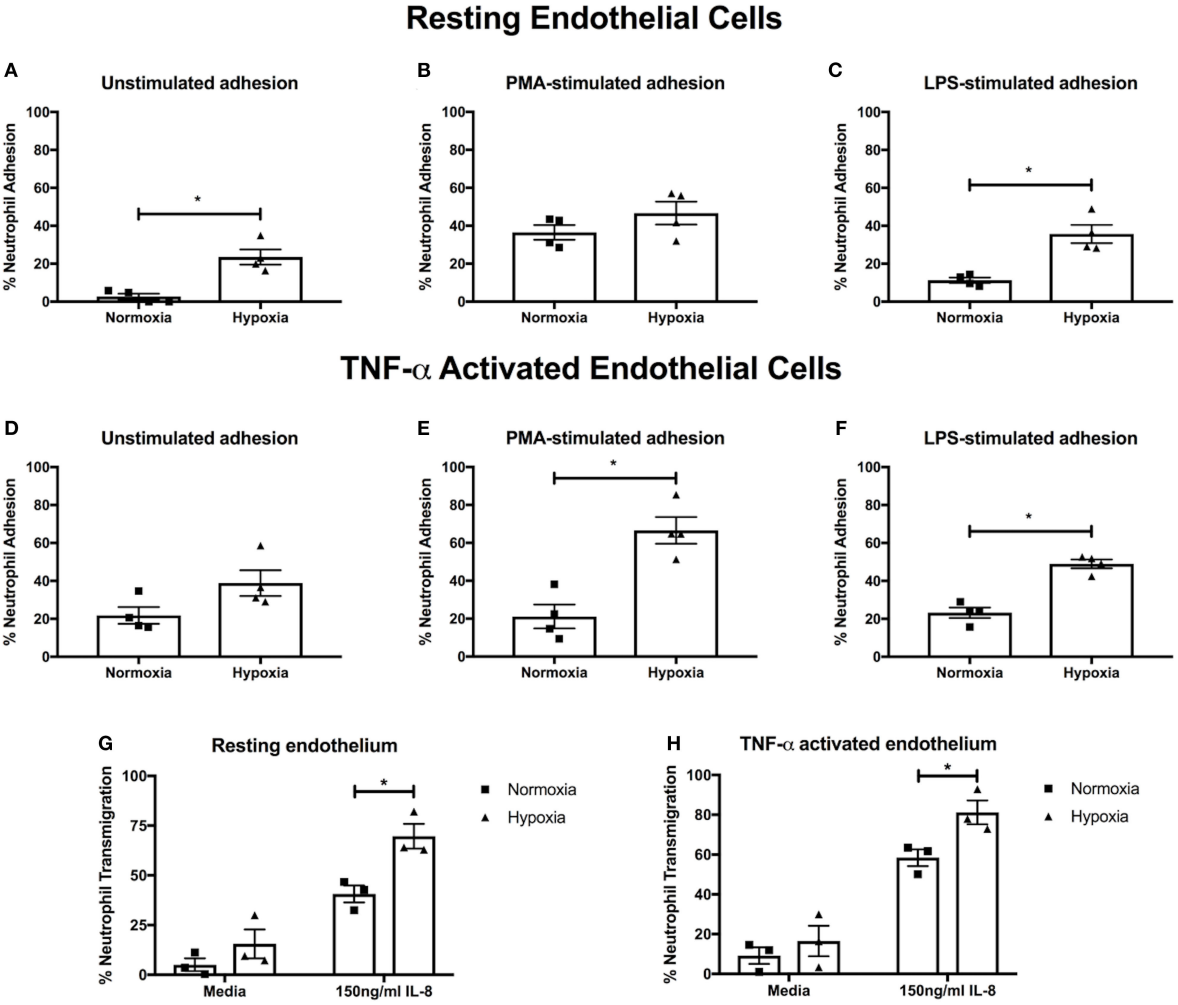
Hypoxia enhances neutrophil adhesion and trans-endothelial migration. BCECF-AM labeled neutrophil adhesion to endothelial monolayers over 30 min was assessed under normoxia (21% oxygen) and hypoxia (1% oxygen). We first examined neutrophil adhesion to resting HUVEC using **(A)** unstimulated neutrophils, **(B)** neutrophils stimulated with 20 nM PMA and **(C)** cells stimulated with 100 ng/ml LPS. Next, we examined the effects of hypoxia upon neutrophil adhesion to activated HUVEC, which had been stimulated with 10 ng/ml TNF-α for 24 h prior to experimentation. We evaluated **(D)** unstimulated neutrophil adhesion, **(E)** neutrophil adhesion in response to 20 nM PMA and **(F)** neutrophil adhesion in response to 100 ng/ml LPS. Finally, the effects of hypoxia upon trans-endothelial migration of CellTracker™ Green labeled neutrophils over 90 min was evaluated. Data are presented as the mean ± SEM from four independent experiments and analyzed by a Wilcoxon matched pairs test. **(G)** Neutrophil transmigration across resting endothelial monolayers was measured under both normoxia and hypoxia in the absence or presence of 150 ng/ml IL-8. **(H)** HUVEC were stimulated with 10 ng/ml TNF-α for 24 h. Neutrophil trans-endothelial migration was subsequently measured in the absence or presence of 150 ng/ml IL-8 under normoxic or hypoxic conditions. Data are presented as the mean ± SEM from three independent experiments and analyzed by two-way ANOVA with a Dunnett's multiple comparison test. ^*^ = *p* < 0.05. HIF, hypoxia-inducible factor; PMA, phorbol 12-myristate 13-acetate.

Having found an effect on NET release, we next examined integrin activation and neutrophil adhesion, which are also implicated in NET induction (39–41). We measured neutrophil adhesion to primary human endothelial cells in the absence or presence of PMA (a general integrin activator) or LPS (to mimic infectious stimuli), stimuli that induce NETs via distinct pathways (42). Hypoxia increased both unstimulated (23.6 ± 4.0% vs. 2.7 ± 1.6%, *p* < 0.05) and LPS-stimulated (35.7 ± 4.8% vs. 11.3 ± 1.4%, *p* < 0.05) adhesion to resting endothelium, whilst PMA-stimulated adhesion, which was already high, was unaffected (Figures 3A–C). We then looked at adhesion to endothelium pretreated with TNF-α, to mimic an inflammatory event. Whilst unstimulated neutrophil adhesion to TNF-α activated endothelial cells was not altered by hypoxia, there was a 3.22- and 2.11-fold increase in PMA- (21.2 ± 6.3% vs. 68.1 ± 8.4%. *p* < 0.05) and LPS-stimulated (23.2 ± 2.8% vs. 49.0 ± 2.3%, *p* < 0.05) adhesion, respectively (Figures 3D–F).

Next, we evaluated neutrophil trans-endothelial migration in the absence and presence of IL-8, which has been shown to induce neutrophil migration. Basal transmigration (in the absence of IL-8) across both resting and TNF-α activated endothelium was unaffected by hypoxia. In contrast, in the presence of IL-8, hypoxia enhanced neutrophil trans-endothelial migration across both resting and TNF-α activated endothelium (*p* < 0.05) (Figures 3G,H).

### Hypoxia Increases Expression of Neutrophil β_2_ Integrins, but Not β_1_ Integrins

**Figure 4:**
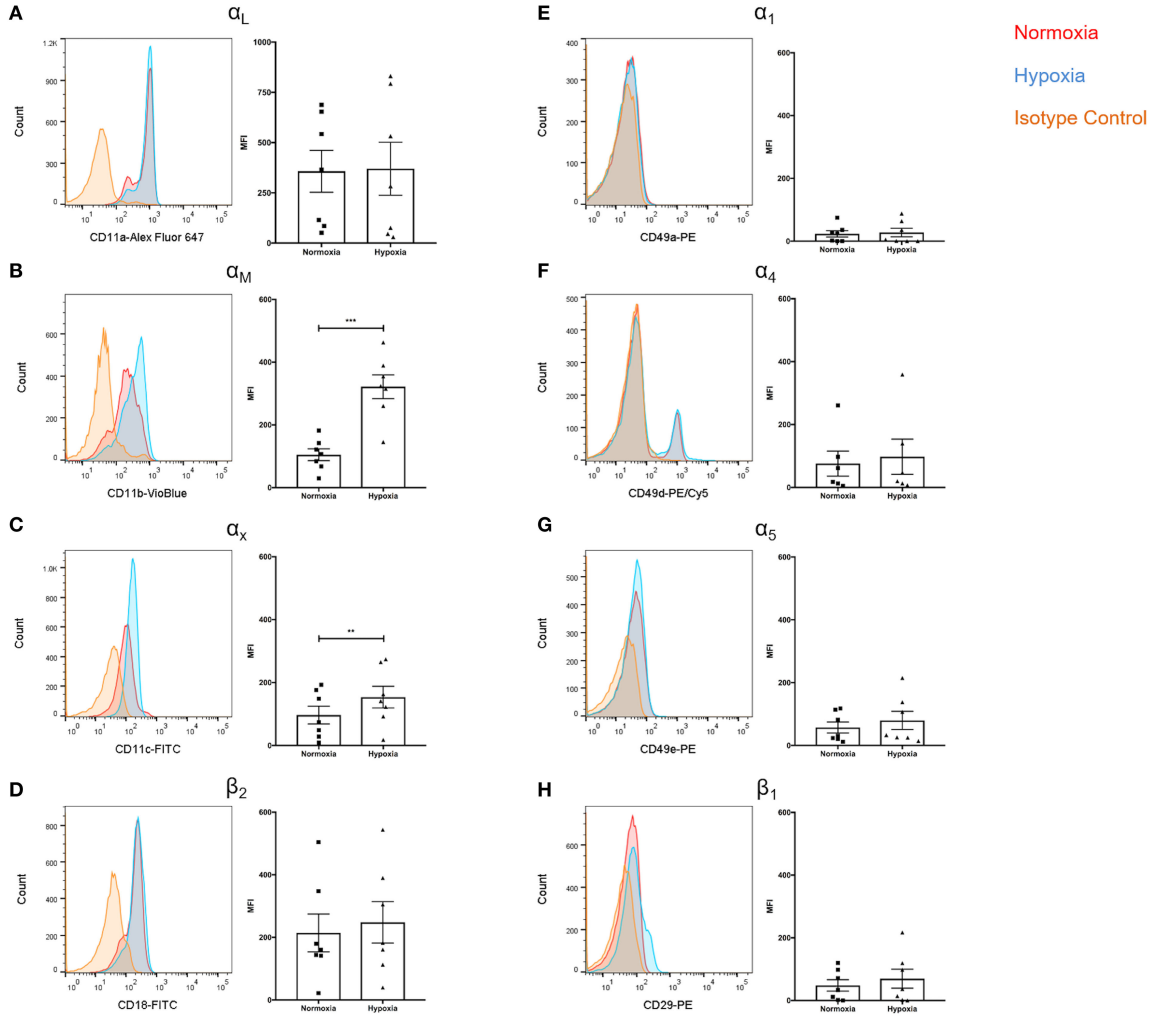
Hypoxia increased neutrophil α_M_ and α_X_ integrin subunit expression. Neutrophil integrin expression was examined following culture under normoxia (21% oxygen) or hypoxia (1% oxygen). Flow cytometry was used to assess expression of the integrin subunits: **(A)** α_L_, **(B)** α_M_, **(C)** α_X_, **(D)** β_2_, **(E)** α_1_, **(F)** α_4_, **(G)** α_5_, and **(H)** β_1_. Data are presented as the mean ± SEM of neutrophils isolated from seven different donors and analyzed by Wilcoxon matched pairs test. ^**^ = *p* < 0.01, ^***^ = *p* < 0.001. HIF, hypoxia-inducible factor.

Given the role of integrins in leukocyte extravasation and reports documenting reduced NETs following integrin blockade (39), we assessed surface integrin expression. Whilst α_L_ expression was unaffected (Figure 4A), significantly higher levels of α_M_ (+3.1-fold, *p* < 0.001) and α_X_ (+1.6-fold, *p* < 0.01) were observed under hypoxia (Figures 4B,C). There were no significant differences in β_2_ expression (Figure 4D). Hypoxia did not have an effect upon α_1_, α_4_, α_5_, or β_1_ integrin subunit expression (Figures 4E–H).

### NET Formation Is Induced by α_M_β_2_ Integrin Activation

**Figure 5:**
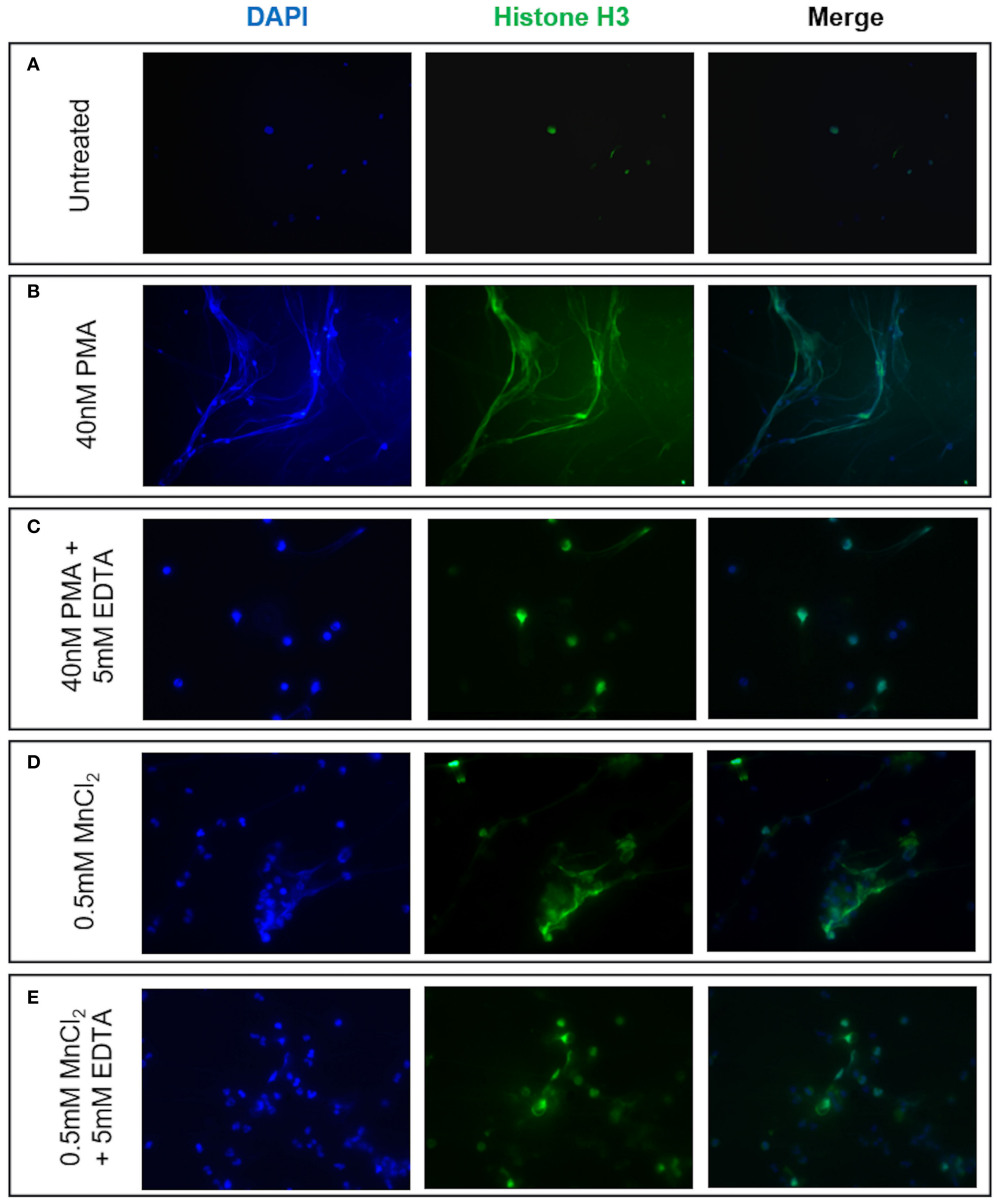
NET formation can be induced by neutrophil integrin activation. The effects of cation-dependent integrin activation upon NET release was examined visually by immunofluorescence staining of both nuclear DNA (DAPI, blue staining) and histone H3 (green staining). **(A)** Untreated neutrophils displayed punctate nuclear staining. **(B)** 40 nM PMA stimulation induced DNA and histone externalization. **(C)** PMA-induced NET release could be mitigated by the addition of 5 mM EDTA (cation chelator). We subsequently examined whether cation-dependent integrin activation could induce NETs. **(D)** Stimulation with 0.5 mM MnCl_2_ induced some DNA externalization. **(E)** DNA externalization was suppressed following cation chelation with EDTA. Representative images are shown from three independent experiments.

**Figure 6:**
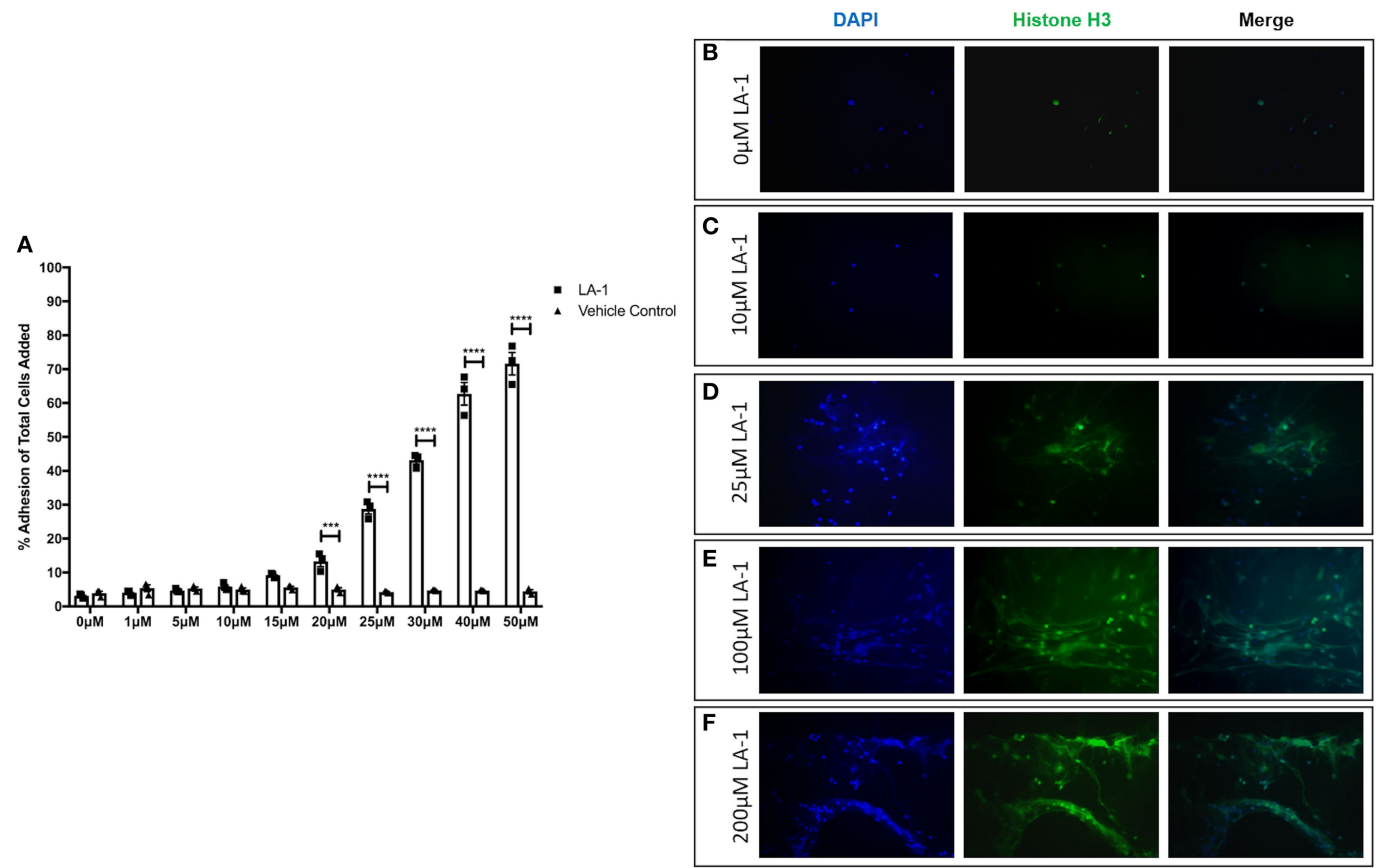
Specific α_M_β_2_ activation induces NET-like structure release. The effects of specific α_M_β_2_ activation upon on neutrophil function by culturing cells with varying concentrations of leukadherin-1 (LA-1). **(A)** Lower concentrations (0–15 μM) of LA-1 did not increased neutrophil adhesion compared to vehicle. From 20 to 50 μM LA-1, we observed a dose dependent increase in neutrophil adhesion. Data are presented as the mean ± SEM from three independent experiments and analyzed by two-way ANOVA with a Sidak's multiple comparison test. The release of NET-like structures was visualized by staining nuclear DNA (DAPI, blue staining) and histone H3 (green staining) in neutrophils. **(B)** Untreated neutrophils and **(C)** neutrophils stimulated with 10 μM LA-1 displayed punctate nuclear staining. **(D)** 25 μM LA-1 stimulation induced greater neutrophil adhesion, however nuclear staining was still punctate. **(E)** Greater numbers of neutrophils were seen following stimulation with 100 μM LA-1 with the addition of externalized DNA and histone staining, forming NET-like structures. **(F)** 200 μM LA-1 stimulation also induced NET releasing neutrophils. Representative images are shown from two independent experiments. ^***^ = *p* = 0.0003, ^****^ = *p* < 0.0001.

Given reports of reduced NET formation following integrin inhibition and our data showing increased α_M_β_2_ and, to a lesser extent, α_X_β_2_ integrin expression, we tested whether integrin engagement induced the release of NETs, using an established model in which neutrophils adhere to fibrinogen. Although there is some base-line adhesion (5.5%), stimulation with PMA increases binding to 83.3% that can be blocked with specific α_M_β_2_ inhibition (Supplementary Figure 1). As expected, no NETs were observed in unstimulated neutrophils, whilst PMA stimulation induced the externalization of histone H3 to form NET-like structures (Figures 5A,B). Cation chelation through the use of EDTA, which abolishes integrin-mediated adhesion, suppressed PMA-induced NET formation (Figure 5C). Finally, global integrin activation by means of manganese chloride treatment, induced some histone H3 externalization that could also be suppressed with EDTA treatment (Figures 5D,E). To confirm the role of α_M_β_2_, neutrophils were stimulated with LA-1, a compound that specifically activates the α_M_β_2_ integrin (43). LA-1 stimulation showed a dose dependent effect upon neutrophil adhesion (Figure 6A). Whilst low concentrations of LA-1 failed to induce the formation of NET-like structures (Figures 6B,C), high levels of LA-1 produced DNA-histone structures similar to PMA-stimulated cells (Figures 6D–F). These results confirmed that α_M_β_2_ integrin activation can induce NETs in human neutrophils.

### Lung Tissue and BAL From Patients With ILD Have More NETs Than Non-ILD Controls

**Figure 7:**
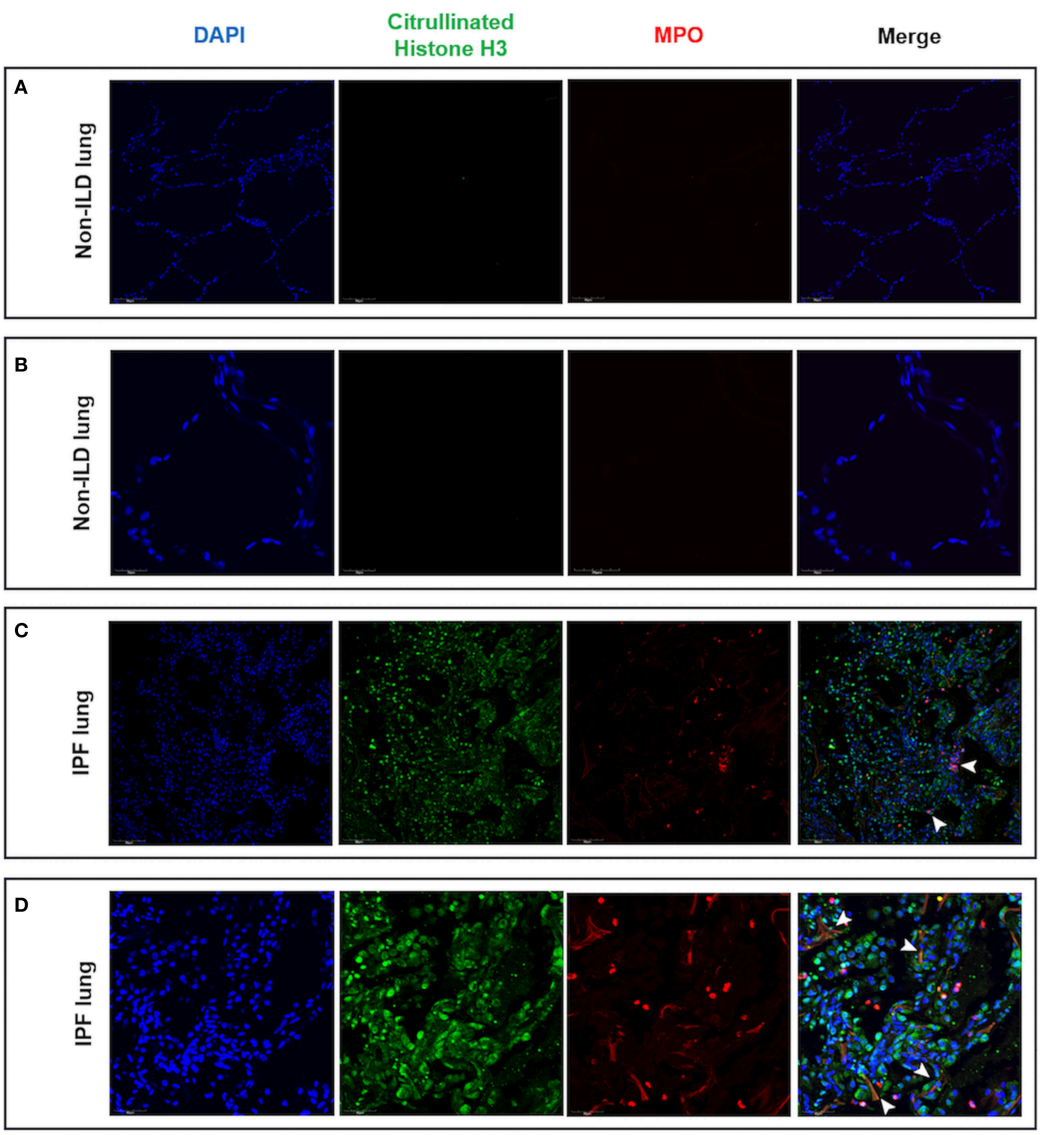
Lung tissue sections from IPF patients contained areas of NET-like structures. Lung tissue from IPF patients and non-ILD control donors was examined for NETs as defined by nuclear DNA (DAPI, blue staining), citrullinated histones (citrullinated histone H3, green staining) and neutrophil-derived proteins (MPO, red staining) by confocal microscopy. **(A)** Low and **(B)** high magnification of non-ILD lung displayed normal lung architecture (seen with DAPI staining) and the absence of cellular infiltrates, histone citrullination and MPO staining. In contrast, **(C)** low and **(D)** high magnification of IPF lung sections showed areas of extracellular DNA release localized with citrullinated histones and MPO, indicating the presence of NET-like structures in fibrotic lung (white arrowheads). Representative images from two non-ILD control or IPF lungs are shown and the size is denoted by the scale bar. ILD, interstitial lung disease; IPF, idiopathic pulmonary fibrosis.

Having found evidence of hypoxia within the ILD lung and shown that hypoxia augments neutrophil activation, we evaluated lung tissue sections for evidence of NETs. In non-ILD control lung sections, we observed normal lung architecture with the absence of cellular infiltrates and lung tissue remodeling at both low and high magnification (Figures 7A,B). In contrast, we noted cellular infiltration in IPF lungs accompanied with the presence of MPO and histone citrullination (Figures 7C,D). Co-localization of extracellular DNA, MPO and citrullinated histones is suggestive of NET formation within the ILD lung.

**Figure 8:**
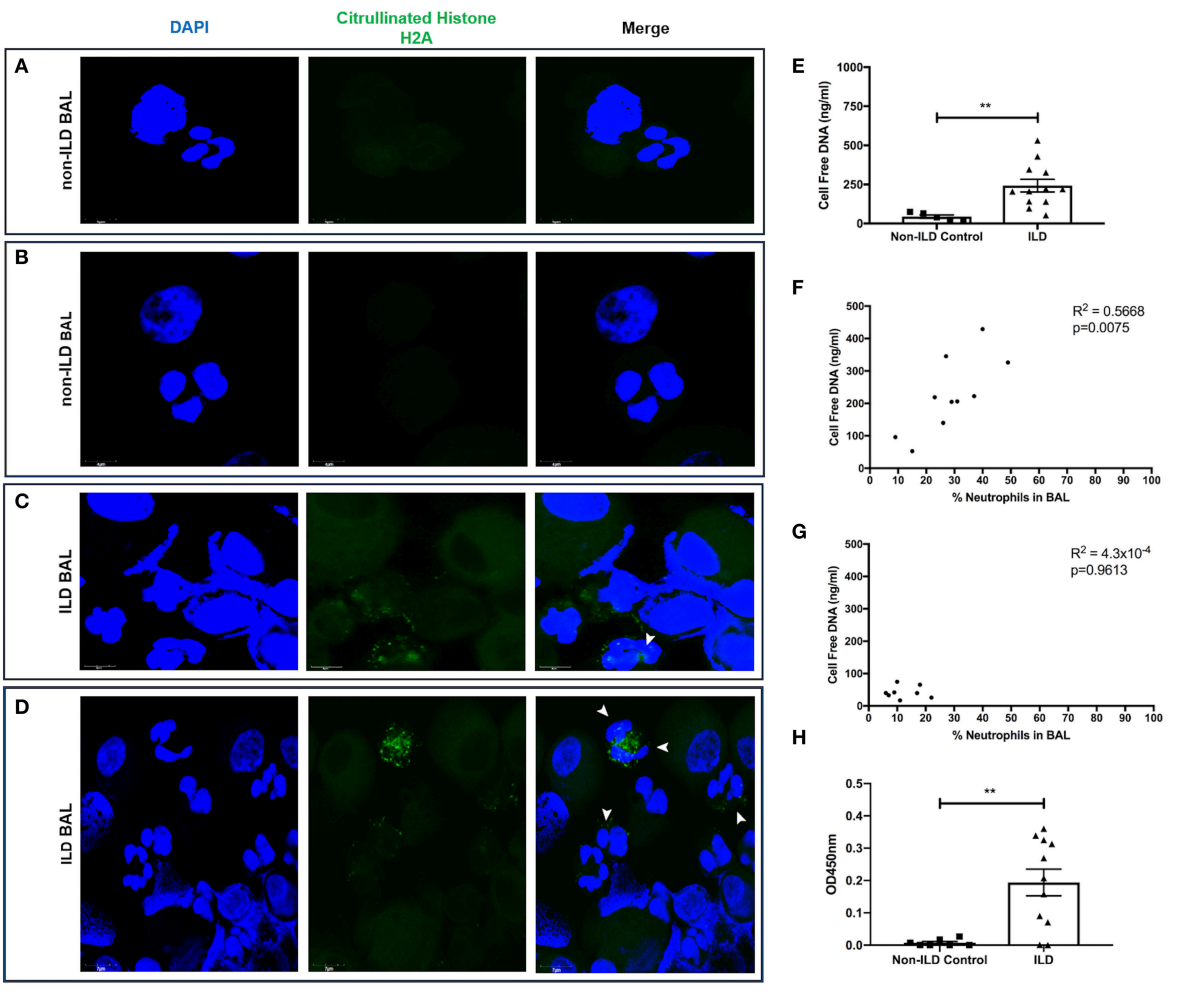
BAL isolated from ILD patients contain more NETs and NET-releasing neutrophils. BAL from ILD patients and non-ILD controls was examined for evidence of neutrophil activation. We first performed confocal microscopy to identify NETs as defined by nuclear DNA (DAPI, blue staining) and citrullinated histone expression (citrullinated histone H2A, green staining). **(A,B)** Non-ILD control BAL cells displayed punctate nuclear staining and lack of citrullinated histones, whilst **(C,D)** BAL cells obtained from patients with ILD showed degrees of DNA externalization from polymorphonuclear cells, along with the presence of citrullinated histones indicative of neutrophils undergoing NET release (white arrowheads). Representative images from two non-ILD or ILD donors are shown and the size is denoted by the scale bar. **(E)** BAL fluid was then assessed for the presence of NETs by Quant-iT™ PicoGreen^®^ dsDNA assay (Thermo Fisher, UK), which found that BAL fluid from ILD patients (*n* = 11) has significantly more cell free DNA compared to non-ILD controls (*n* = 8). From differential cell counts, we found that **(F)** cell free DNA positively correlated with the proportion of BAL neutrophils in patients with ILD, **(G)** but not in non-ILD controls. **(H)** Finally, we also tested BAL fluid using an optimized capture ELISA detecting MPO-citrullianted histone H3 complexes, demonstrating that BAL fluid from ILD patients (*n* = 11) has significantly more NETs compared to non-ILD controls (*n* = 8). Data were analyzed by either a Mann-Whitney test or two-tailed Pearson correlation coefficients. ^**^ = *p* < 0.01. BAL, bronchoalveolar lavage; ILD, interstitial lung disease.

Finally, we examined BAL for evidence of neutrophil activation. We generated slides with BAL and stained with DAPI and citrullinated histone H2A to identify the presence of neutrophils initiating the production of NETs. Control BAL neutrophils displayed punctate DAPI staining with the absence of citrullinated histones (Figures 8A,B). In contrast, we observed the presence of citrullinated histones and more diffuse DNA staining in BAL polymorphonuclear cells obtained from patients with ILD, indicative of neutrophils forming NET-like structures (Figures 8C,D). We then obtained BAL from 11 ILD patients (ILD-BAL) and seven non-ILD controls (control BAL) and quantified levels of cell-free DNA. We found ILD-BAL had 5.5-fold greater cell-free DNA content compared to control BAL (*p* < 0.01) (Figure 8E). Cell-free DNA content positively correlated with neutrophil counts (% of total cells) isolated from ILD-BAL (*p* = 0.0075) (Figure 8F), but not in control BAL (Figure 8G). To verify that these were NETs, we also examined BAL for the presence of MPO-citrullinated histone H3 complexes. Similar to total cell-free DNA, we observed significantly greater values in ILD-BAL (+21.9-fold, *p* < 0.01), indicating greater levels of NETs (Figure 8H).

## Discussion

Neutrophil dysfunction and aberrant activation have been implicated in the pathology of numerous diseases including autoimmune rheumatic diseases (44–48) and cancer (49–52). More recently the release of NETs, essential for robust immune defense against pathogens, has also been linked to increased immunopathology in patients with COVID-19, a disease characterized by neutrophilic inflammation and endothelial activation (53, 54). The precise mechanism in which neutrophils contribute to ILD pathogenesis is unknown. Early work from the 1980s began to explore whether neutrophils might contribute to IPF pathology (11–14), however this avenue of research lost momentum. Since then, reports have associated increased neutrophil migration and activation with severe pulmonary disease both in animal models (22, 55) and man (16, 19, 20). We report, for the first time, that neutrophils and endothelial cells in ILD lung biopsies display HIF-1α expression and provide evidence of the extracellular release of nuclear DNA, citrullinated histones and MPO, indicative of NET formation in the ILD lung. Given the profound effects hypoxia exerts upon neutrophil survival and function (26, 27), these findings led us to investigate whether hypoxia affects neutrophil extravasation and activation, thus contributing to ILD pathology.

Integrins are adhesive molecules that enable leukocytes to interact with their external environment. Similar to a previous report (56), we found increased β_2_ integrin expression in neutrophils under hypoxia, but specifically found increased α_M_ and, to a lesser extent, α_X_ integrin subunit expression. Interestingly, the α_M_β_2_ and α_X_β_2_ integrins also function as complement receptors, which may be relevant to ILD pathology given that increased levels of complement C3a and C5a and roles for their receptors have been reported in IPF (57, 58). Moreover, studies using the bleomycin-induced mouse model of IPF highlight roles for both C3 and C5 in pulmonary fibrosis (59, 60). Upregulation of β_1_ integrins has been described under hypoxia (61), however, there are no reports assessing expression in neutrophils. A lack of effect may be explained by the relatively low β_1_ integrin expression in human neutrophils. Taken together, the evidence indicates that neutrophils predominately engage via β_2_ integrins, a mechanism which is enhanced under hypoxia.

Early studies demonstrated that hypoxia enhances neutrophil adhesion to endothelial cells (62), epithelial cells (63), and trans-epithelial migration (64). In support of these findings, our results show altered function of healthy neutrophils with increased neutrophil adhesion and trans-endothelial migration under hypoxia. In addition, we report that hypoxia enhances NET formation. Given that the gold standard markers or methods for the induction and detection of NETs have not been established (65), we used several different techniques to confirm the release of NETs by cultured neutrophils: the co-localization of nuclear DNA and histone H3 complexes by immunostaining; confocal imaging of DNA, citrullinated histones and MPO; quantification of cell-free DNA; and the detection of neutrophil-derived proteins (MPO) and citrullinated histone H3 complexes. Our observations complement studies in the literature showing that pharmacological HIF-1α stabilization enhances NET release and inhibition of HIF-1α reduces NETs and bactericidal activity (28, 29).

Whilst HIF-1α stabilization has been shown to promote NET production (28), the opposing effect of hypoxia upon NET formation has also been described. In contrast to our findings, Branitzki-Heinemann et al. found hypoxia reduced levels of both spontaneous and PMA-induced NET release (66). Whilst the definitions of hypoxia and normoxia were identical (1% oxygen vs. 21% oxygen), key methodological differences may explain the contrasting conclusions. Branitzki-Heinemann et al. isolated neutrophils using gradient centrifugation with PolymorphPrep, whilst this study used Percoll PLUS. Whilst a minor difference, comparative analysis of isolation procedures found reduced CD15 and CD66b expression in neutrophils isolated with PolymorphPrep (67), which may have further implications on *ex vivo* function. Both reports used PMA to initiate NET formation, however at different concentrations. In this study, neutrophils were stimulated with 40 nM PMA for 4 h, whilst the earlier Branitzki-Heinemann study treated cells with 25 nM PMA for 3 h. It is possible that stimulating neutrophils with a higher concentration of a potent PKC activator may account for the differing response to hypoxia. Finally, when quantifying NETs, Branitzki-Heinemann et al. seeded neutrophils on coverslips coated with poly-L-lysine whilst neutrophils in this study were exposed to either nunclon-treated or fibrinogen-coated surfaces. This difference may result in varying degrees of α_M_β_2_ engagement and alter neutrophil responses.

Our data suggest that α_M_β_2_ engagement may induce NET formation, which is supported by recent work demonstrating α_M_β_2_ triggering NET release (68). Moreover, several studies have shown that α_M_β_2_ blockade reduced NET release (39, 40, 69, 70), which indirectly supports our work. Our findings that α_M_β_2_ interaction with ligand may regulate NET formation builds on earlier findings, which showed that PMA stimulation led to high levels (>80%) of chromatin decondensation (a prelude to NET formation) and was not affected by substrate (41). In contrast, LPS stimulation led to lower basal levels of decondensation (~20%) and levels increased with matrix stiffening and increased cell spreading on β_2_ and β_1_ integrin ligands. This effect was inhibited by PI3K inhibition suggesting a dependence on integrin outside-in signaling. The impact of matrix stiffness is highly relevant in fibrotic ILD as lung fibrosis changes tissue elasticity. There are some methodological differences between this work and that of Erpenbeck and colleagues. In particular, our neutrophils were rested in hypoxia or normoxia overnight before PMA stimulation. Our background NET levels were lower in response to PMA rather than the dramatic increase from <5 to >80% previously reported. It is possible that when the level of NETs is lower, integrin activation related to matrix stiffness plays more of a role regardless of the stimulus. This finding further emphasizes the importance of a complete description of the experimental system (65). This finding may have particular relevance not only to ILD but also to other fibrotic lung diseases. The finding that hypoxia drive NET formation may also be of relevance to non-fibrotic pathologies including COVID-19, a disease characterized by severe hypoxia, NET release and hyper-inflammation (53, 71).

Further work could build on the level of hypoxia required to produce these effects. In our study we used 1% oxygen, however, normal oxygen levels can range from 5.0 to 13.2% in the circulation and 0.5 to 2.7% in tissues (72, 73). Further experiments could determine whether lesser degrees of hypoxia, seen in clinical practice, also enhance neutrophil adhesion, trans-endothelial migration and NET formation. These studies could determine the relative importance of HIF transcription factors to NET formation and release, through the use of previously identified small molecule inhibitors (74–76), and better understand the relationship between neutrophil activation and hypoxia.

We observed tissue-specific HIF-1α expression in ILD lung tissue, mainly restricted to pulmonary endothelial cells and neutrophils, with only minimal upregulation in areas of epithelium and fibrosis, which may hold pathological relevance. Previously, markers of hypoxia have been variably reported in the epithelium of patients with IPF. Several authors have found HIF-2α and CA-IX within the IPF fibrotic reticulum and HIF-1α in the overlying epithelium with IHC (7), [albeit sometimes in a single patient (8)]. HIF-1α is more readily found in the mouse bleomycin model of PF raising the question of differences between the two species and insults (77). Whilst epithelium-specific HIF-1α deletion has no effect upon radiation-induced enteritis, mice with endothelium-specific HIF-1α deletions present with reduced intestinal damage (78). HIF-1α is known to contribute to the pathology of pulmonary hypertension (79, 80), with some work specifically interrogating endothelial HIF signaling (81). Neutrophilic inflammation has also been associated with pulmonary hypertension (82), and believed to drive angiogenesis via NET release (83). Therefore, the pulmonary pathology in ILD patients may in part be attributed to endothelial and neutrophil HIF-1α expression enhancing neutrophil recruitment and NET formation within the lung. In this paradigm, enhanced NETs would initiate angiogenic signals and drive lung pathology. Interestingly, the model of neo-angiogenesis underlying ILD pathology has attracted interest, and the powerful angiogenic inhibitor, nintedanib, shown to have therapeutic benefits in a range of fibrotic ILDs (84) and endothelial reactivity with angiogenesis is also noted in COVID-19.

Our findings of hypoxia driving NET formation complements the increasing evidence that NETs may play a role in many acute and chronic lung diseases (85), including ILD by stimulation of fibroblasts (17). In PF, we propose that elevated NET release may cause epithelial cell damage, dysfunction and death, drive innate and adaptive immune cells activation, and promote a pro-fibrotic environment that ultimately facilitates the progression of pulmonary fibrosis. For our experiments, we used neutrophils isolated from peripheral blood donated from healthy volunteers. Further work could examine cells isolated from patients with ILD, isolated from either peripheral blood or BAL, to determine whether hypoxia has similar or an enhanced effect within this patient population. In addition, it would also be interesting to examine neutrophil function using autologous human serum from patients with ILD to provide further insight under more physiological conditions *in vitro*.

Hypoxia is also thought to have differential integrin-independent effects upon NET formation (66). Further experiments exploring the effects of hypoxia upon neutrophil activation by examining neutrophil responses to a wider range of stimuli, such as bacterial/fungal antigens, ionomycin or monosodium urate crystals, which also activate β_2_ integrins (86), may therefore provide further insight into the relationship between neutrophil function, integrin activation and hypoxia. Interestingly, the recent consensus article written by opinion leaders to present prevailing concepts and state of the science in NET-related research and elaborate on open questions and areas of dispute does not specify which stimulus should be used to induce NET formation (65). Instead, this consensus article suggests that researchers should specify in detail culture conditions, including base medium, use of serum, absence of platelets and surface constitution of the cell culture plate, as well as stimulus and source/preparation of inducer used. Our analysis of NET formation ultimately focuses on the end stages culminating in NET release into cell supernatants. Further work could explore the effects of hypoxia upon the preceding stages such as cell spreading and chromatin decondensation/nuclear swelling (41, 87), to better understand how hypoxia affects the entire NET formation process.

In conclusion, we report that the ILD lung contains molecular features of hypoxia, mainly localized to neutrophil and endothelial cells, which may contribute to disease pathology. Hypoxia enhanced neutrophil β_2_ integrin expression, which translated to augmented adhesion and migration across endothelial cells, and NET release. Our findings are further supported by demonstration of NETs within the human fibrotic lung seen through imaging of IPF lung sections and BAL cells, as well as detecting cell-free DNA and MPO/citrullinated histone complexes in BAL obtained from patients with ILD. Taken together, our work begins to elucidate a potential role of hypoxia in driving neutrophil recruitment and activation within the airspace to promote a pro-fibrotic environment. These findings offer a rationale for future translational medicine exploration of a novel neutrophil HIF-1α-integrin axis as a potential therapeutic target in fibrotic ILD.

## Data Availability Statement

All datasets generated for this study are included in the article/Supplementary Material.

## Ethics Statement

The studies involving human participants were reviewed and approved by UK & National Research & Ethics Committee. The patients/participants provided their written informed consent to participate in this study.

## Author Contributions

AK designed and performed experiments, analyzed the data, and contributed to writing the manuscript. DC, JS, and HB obtained and analyzed BAL samples and performed microscopy. TM performed lung cyrobiopsies that were used for lung staining. SK and MR-J performed and analyzed IHC images. CP and VR contributed to experimental design and analysis. IG and JP conceived the study, designed the experiments, were involved in data analysis, and contributed to the writing of the manuscript. All authors contributed to the article and approved the submitted version.

## Conflict of Interest

The authors declare that the research was conducted in the absence of any commercial or financial relationships that could be construed as a potential conflict of interest.
