# Supplementary material for: Identification of a Novel HIF-1α-αMβ2 Integrin-NET Axis in Fibrotic Interstitial Lung Disease
Source: Front Immunol. 2020 Oct 15;11:2190. doi: 10.3389/fimmu.2020.02190 (PMC7594517; doi:10.3389/fimmu.2020.02190)
Supplement: Supplementary file 1 [file Data_Sheet_1.DOCX]

**Supplementary Material**

| **Sample** | **Differential Cell Count (%)** | | | |
| --- | --- | --- | --- | --- |
|  | **Macrophage** | **Neutrophil** | **Lymphocyte** | **Eosinophil** |
| ILD01 | 59 | 29 | 5 | 6 |
| ILD02 | 47 | 27 | 8 | 19 |
| ILD03 | 47 | 40 | 3 | 10 |
| ILD04 | 35 | 49 | 10 | 7 |
| ILD05 | 62 | 23 | 2 | 14 |
| ILD06 | 79 | 9 | 12 | 1 |
| ILD07 | 59 | 31 | 8 | 2 |
| ILD08 | 66 | 26 | 4 | 5 |
| ILD09 | 69 | 26 | 4 | 1 |
| ILD10 | 60 | 37 | 2 | 0 |
| ILD11 | 68 | 22 | 1 | 8 |
| Non-ILD01 | 72 | 22 | 3 | 3 |
| Non-ILD02 | 81 | 11 | 8 | 0 |
| Non-ILD03 | 91 | 6 | 2 | 2 |
| Non-ILD04 | 92 | 6 | 3 | 0 |
| Non-ILD05 | 78 | 18 | 0 | 3 |
| Non-ILD06 | 86 | 10 | 3 | 1 |
| Non-ILD07 | 97 | 3 | 0 | 0 |

**Table S1. Cellular compositions of bronchoalveolar lavage (BAL) fluid.** BAL fluid was obtained from patients undergoing diagnostic bronchoscopy. BAL cells were isolated by centrifugation, differentially stained with a Rapid Romanowsky Stain Kit (TCS Biosciences Ltd, UK) and counted using a conventional brightfield microscope.


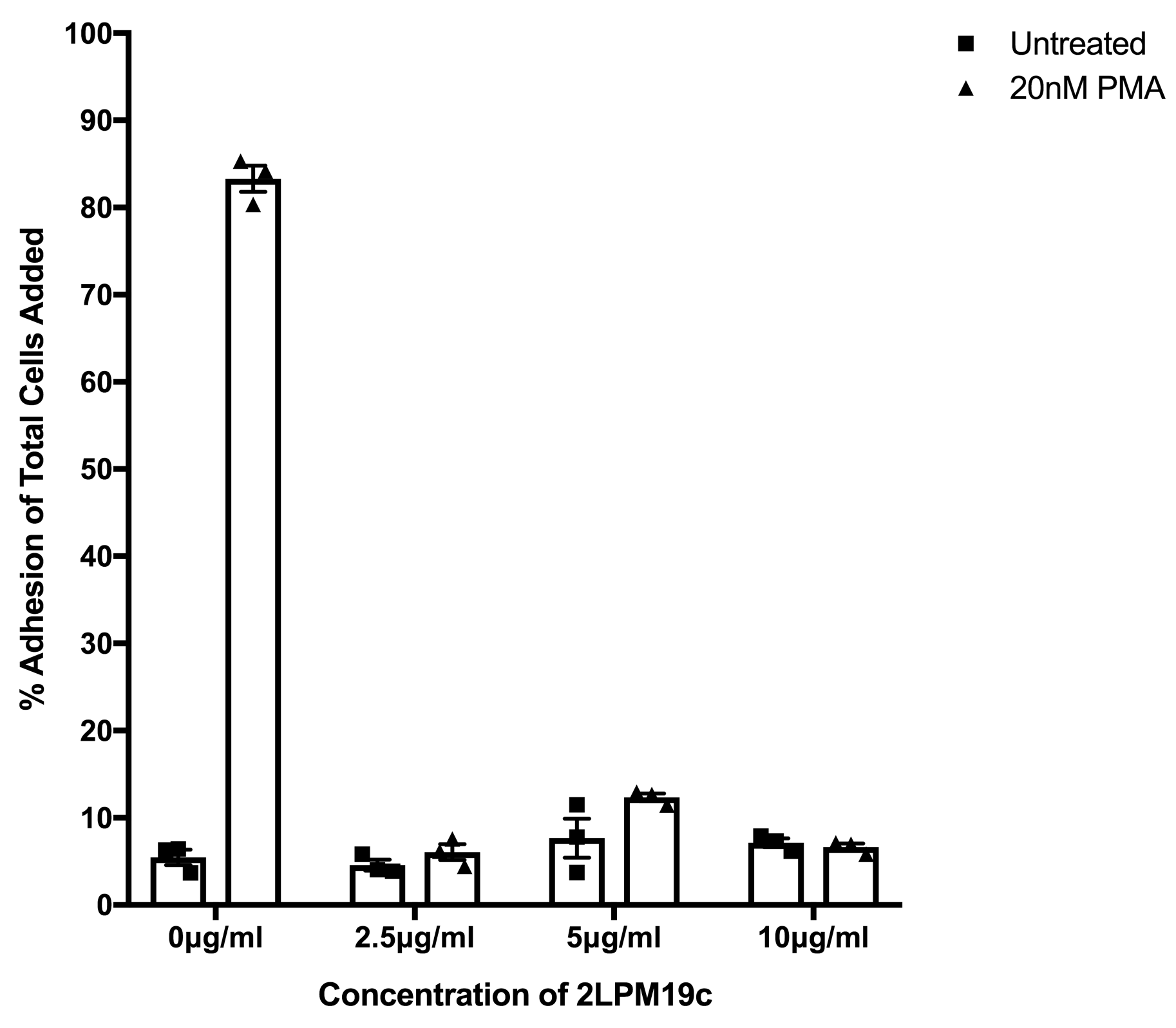


**Figure S1. PMA-induced neutrophil adhesion to immobilised fibrinogen can be mitigated by α_M_β_2_ inhibition.** Neutrophil adhesion was assessed in the absence or presence of 20nM PMA with varying concentrations of the α_M_β_2_-specific blocking antibody 2LPM19c. Adhesion was blocked from 2.5μg/ml 2LPM19c, however we still observed low levels of α_M_β_2_-independent adhesion in both untreated and PMA-stimulated cells.
